# Quantifying the Risk of Localised Animal Movement Bans for Foot-and-Mouth Disease

**DOI:** 10.1371/journal.pone.0005481

**Published:** 2009-05-08

**Authors:** David Schley, Simon Gubbins, David J. Paton

**Affiliations:** Institute for Animal Health, Pirbright Laboratory, Surrey, United Kingdom; University of Swansea, United Kingdom

## Abstract

The maintenance of disease-free status from Foot-and-Mouth Disease is of significant socio-economic importance to countries such as the UK. The imposition of bans on the movement of susceptible livestock following the discovery of an outbreak is deemed necessary to prevent the spread of what is a highly contagious disease, but has a significant economic impact on the agricultural community in itself. Here we consider the risk of applying movement restrictions only in localised zones around outbreaks in order to help evaluate how quickly nation-wide restrictions could be lifted after notification. We show, with reference to the 2001 and 2007 UK outbreaks, that it would be practical to implement such a policy provided the basic reproduction ratio of known infected premises can be estimated. It is ultimately up to policy makers and stakeholders to determine the acceptable level of risk, involving a cost benefit analysis of the potential outcomes, but quantifying the risk of spread from different sized zones is a prerequisite for this. The approach outlined is relevant to the determination of control zones and vaccination policies and has the potential to be applied to future outbreaks of other diseases.

## Introduction

Foot-and-Mouth Disease (FMD) remains of enormous socio-economic importance, both in disease-free countries and where it is endemic. Due to its highly contagious nature and economic importance, FMD is the first disease for which the OIE (World Organisation for Animal Health) lists a country's status. In addition to the impact of FMD on animal welfare and productivity, the loss of disease-free status results in costly trade restrictions. Its economic impact – as witnessed in the UK in 2001 (Thompson et al., 2002) - is considered sufficient to justify stringent control measures including the implementation of a stamping-out policy with culling of animals on infected farms and, in certain circumstances, where infection is suspected rather than confirmed.

An important part of any containment and eradication policy is the imposition of movement restrictions for susceptible livestock. There was severe criticism of the delay in February 2001 in bringing this into effect within the UK, with analysis (Ferguson et al., 2001), (Woolhouse et al., 2001) showing that a significant reduction in the basic reproduction ratio of infected premises (IPs) was achieved following the imposition of a national movement ban, in part due to the initial widespread and undiagnosed transmission - the “silent spread” period (Anderson, 2002); we define the basic reproduction ratio as the number of new IPs per existing IP.

Such a policy has major practical and economic implications. The inability to take livestock to market or slaughter at an appropriate time incurs direct and indirect losses: in 2001 a significant proportion of animals - at least two and a half million out of more than six and a half million (Haydon et al., 2004) - were culled for welfare reasons, after being stranded on farms without appropriate food and facilities, rather than because of evidence of infection. Losses estimated at £100 million (National-Farmers'-Union, 2007) resulted from national restrictions imposed in response to an FMD outbreak in 2007 in the UK that was contained at a relatively local level (Defra, 2007b), dwarfing the £1.5 m estimated to have been lost by those farms directly involved (National-Farmers'-Union, 2008). It is therefore appropriate to ask whether such stringent restrictions are necessary in parts of the country at a significant distance from affected areas or if it is possible to consider a regional policy instead.

In the European Union (EU) the minimum size for a Control Zone (CZ) around any FMD outbreak is 10 km radius, comprising an intensively monitored inner Protection Zone (PZ) of at least 3 km radius and an outer Surveillance Zone (SZ) (Anonymous, 2003). Regionalisation, whereby movement restrictions are enforced more widely is also recommended where FMDV appears to be spreading despite the imposition of other measures (Anonymous, 2003). Here, we evaluate regionalisation and the imposition of a Restriction Zone (RZ) around any IP and its obligatory CZ. At present we do not consider the imposition of any additional measures in such zones beyond the maintenance of a ban on the movement of susceptible animals after it has been lifted nationally.

Given the potential for rapid spread in unrestricted areas we focus on the risk of the disease escaping any imposed RZ: that is, spreading to a premise outside the zone. Only by quantifying this risk is it possible to evaluate the economic benefits of being able to trade animals (and take them to slaughter) against the significant costs of the disease spreading faster and further; commensurately, we acknowledge that this analysis can only be applied in the context of an economic understanding.

## Results

**Figure 1:**
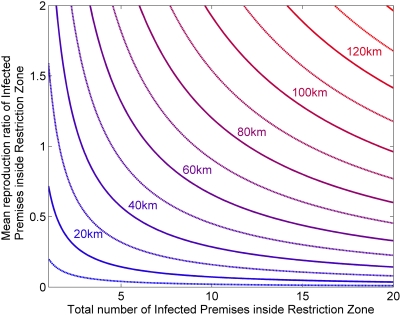
Required Restriction Zone radii to maintain the risk of escape below 1%, given the number of IPs 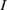 and the mean reproduction ratio 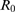 of all IPs within the zone.

Figure 1 shows the required radius for a RZ containing 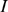 IPs with mean reproduction ratio 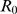 to maintain the risk of escape (infection of premises outside of the RZ) from primary transmission below 1%. Secondary transmissions can be included but have a relatively small effect on the required radius for realistic values of 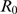.

The risk of escape increases exponentially with 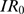 so that the policy decision of what risk level is deemed acceptable will quickly determine whether local RZs are appropriate. An epidemic of the size of that experienced in the UK in 2001 - totalling over 2000 IPs and peaking at over 50 reported cases per day [1], [2] - would generate sufficiently large and dispersed RZs for regionalisation not to be a practical option until considerably later in the outbreak.

The 2007 outbreak of FMD in the UK began with a small cluster of two IPs [3] that triggered a national movement ban and the imposition of a 10 km Control Zone [4], , in accordance with EU regulations [5],. Shortly after restrictions where lifted a second cluster of IPs was found [6], resulting in their reinstatement, although it is clear that transmission occurred between farms while the movement band was still in place [7], [8]. Assuming the original IPs had a mean reproduction ratio of 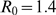 (equivalent to the situation in the UK on 24th February 2001 [9] after restrictions had been put in place) the predicted risk of escape from a 10 km RZ is 13%: for a risk of less than 1% a RZ of 40 km would have been required. Exactly what level of risk is acceptable will depend upon a cost-benefit analysis of relaxed restrictions versus the consequences of further outbreaks.

**Figure 2:**
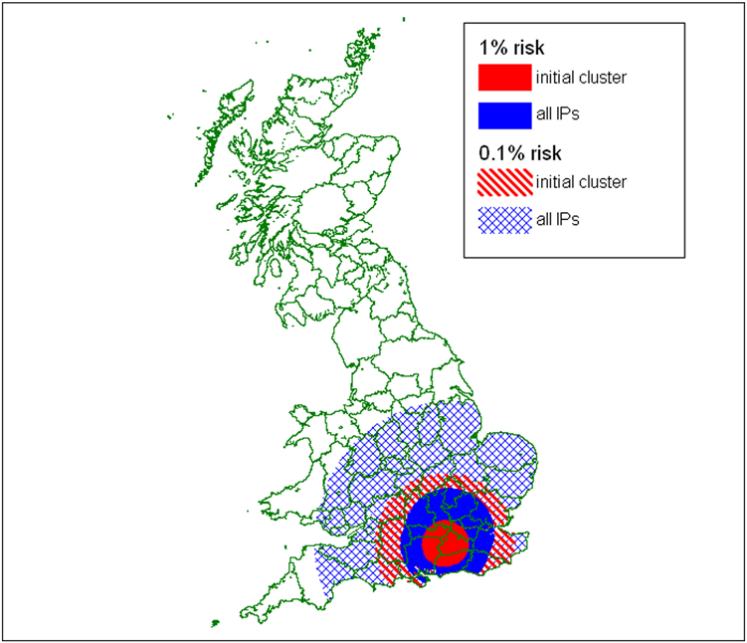
Suggested Restriction Zones with an escape risk of less than 1% and 0.1%, following the Foot-and-Mouth outbreak in the UK in 2007 (based on a predicted mean basic reproduction ratio).

For a risk of further escape from the second cluster of below 1% a 67 km RZ would have been required, but this encompasses the first cluster which – if still considered a risk (due to incomplete tracing for example) – would have required a combined RZ of 77 km. An acceptable risk of only 0.1% dramatically increases the RZ sizes required, to 120 km (initially) and 229 km (following the second cluster); the various RZs are shown in Figure 2. Although such a precautionary approach rapidly results in the imposition of a movement ban over a large area, it is also true that a substantial proportion of the country remains unrestricted. For comparison we note that in response to the bluetongue outbreak in the UK a 150 km Protection Zone was established out of which untested-animal movements were not allowed [10].

The sensitivity of the RZ radius, 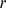, to changes in the transmission kernel parameters 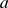 and 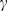, was evaluated by calculating changes in the RZ radius for values of the parameters in their 95% confidence intervals. Changes in the radius, 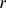, with respect to 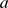 and 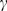, are proportional to 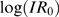 resulting in a maximum increase (decrease) in the RZ radius required for 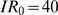 of 34% (24%). In terms of the 2007 clusters, the fitted model has a confidence interval of: (26 km, 63 km) for a 40 km RZ; and of (48 km, 132 km) for a 77 km zone. As discussed in the main section, results are highly sensitive to the choice of acceptable risk 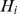, which is the main determinant of the RZ radius.

## Discussion

The evaluation of RZs should always be supplementary to primary risk appraisal involving the tracing of animal movements, known trade routes and other infection pathways from an IP. The automatic imposition of a nationwide movement ban - long enough to cover the “silent spread” period [11] - is necessary in order for this to be carried out and prevent further spread, although potential but unconfirmed locations could always be included in any RZ analysis as a precaution. During this time an assessment of the likely basic reproductive ratio of any cluster of IPs could be made, which would be dependent not only on the virus strain but also on regional factors such as farm-types and densities. Previous studies e.g. [9] have calculated significantly different 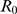 values for different regions during an outbreak, including the generation of 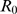 maps [1]: thus different sized RZs for similar sized outbreaks may be justified in different parts of the country. More importantly, this delay would allow for a cost-benefit analysis and stakeholder consultation, necessary to support any policy of regionalisation, to be undertaken.

The critical determinant of the required size of the RZ is the transmission kernel. A number of transmission kernels for individual farm-to-farm transmissions have been published for outbreaks such as that in the UK in 2001, including a historic kernel [1] limiting all transmissions to the furthest recorded in 2001 (i.e. below 60 km), and an extended kernel [12], limiting transmission to below 80 km (though this latter kernel was not designed to incorporate airborne spread). Clearly, using either of these kernels would result in RZs of radius 60 km and 80 km, respectively. Moreover, they would predict that the risk of escape from the RZ was zero. Consequently, using a fitted distribution (as was the case in this study) yields a more conservative estimate for the radius of the RZ, because such an approach allows for low probability, long-distance transmission events.

**Figure 3:**
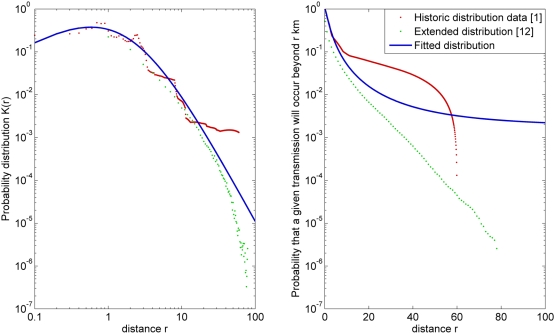
The probability of a given transmission occurring (left) at a given distance and (right) beyond a specified distance for the model transmission distributions and those based on the historic transmission kernel [1], and an extended transmission kernel [12], derived from the 2001 UK outbreak.

In comparison with distributions based directly on published historic kernels [1] or extended kernels [12] the model probability distribution generates the intermediate value for transmission beyond 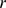 from below 4 km up to beyond 50 km (figure 3). The historic kernel restricts all transmissions to the furthest recorded distances, below 60 km, while the extended does not allow for transmissions beyond 80 km. Only 0.39% of transmissions occur above 50 km (model), as opposed to 1.4% (historic) and 0.016% (extended) respectively.

It is difficult to say what effect the application of RZs would have had on past outbreaks since epidemics are highly dynamic, making the timing of actions critical. Nevertheless, for outbreaks with long tail end periods, such as that in the UK in 2001, which are highly localised, a regional policy offers a clear intermediary step between a national ban and complete relaxation of rules outside of CZs. Given that transmissions from known IPs during the 2007 UK outbreak occurred during the times that a national movement ban was in place it is unlikely that a RZ policy alone would have affected the course of the epidemic. Evaluation of the expected risks of transmission under such conditions could, however, have informed the formulation of Control and/or Surveillance Zones beyond the mandatory 10 km. The imposition of a 40 km zone around the initial cluster, designed to capture all subsequent transmission with 99% probability, would have contained all ensuing IPs. As a SZ this should have led to the detection of the second cluster prior to any relaxation of restrictions (although this would also have generated a substantial increase in the amount of serosurveillance required); as a RZ it could have been kept in place for some time after the national movement ban had been lifted.

Because the model only relies on epidemiological abstractions it can, in theory at least, be easily applied to other diseases. In practice, however, we are limited by requiring a good understanding of both the transmission distribution and the basic reproductive number of sources, as provided for foot-and-mouth in the UK by the 2001 outbreak.

In any future outbreak, in the EU at least, the use of vaccination will be considered as part of any control and eradication programme. This will most likely take the form of reactive (local) vaccination, although there is still a debate over what the best protocol would be [13], [14], [15]. While the implementation of RZs as opposed to a national movement ban is unlikely to require additional resources, it is also important that such a policy does not conflict with other control measures. Results given here have already indicated that any RZs would comfortably include existing CZs and SZs. Comparison with suggested optimal vaccination zones (VZs) – based on resource limitations - show that these would also be within any RZ with an escape probability of 1% or less: for example, for all the different vaccination strategies considered by [13] the 95% confidence limit for the optimal ring size radius was always below 23 km (c.f. Figure 1). The only exception to this is the suggested strategy of vaccinating at capacity regardless of the outbreak size, prioritising farms purely by proximity to existing IPs, which means that there is no limit to the distance at which vaccination occurs. It is difficult to see how or why this policy would be adopted in practice, if only for trade considerations, without the establishment of regions from which exports were prohibited and outside of which vaccination would not be undertaken: something which could be achieved through the creation of RZs. The definition of RZs is, therefore, completely compatible with the establishment of VZs as well as CZs and SZs, and could be used to inform or at least complement them, thereby helping to minimise subsequent trade restrictions.

While we have considered localised movement bans here, this should in no way suggest anything but the highest levels of vigilance and bio-security precautions being adhered to everywhere during an outbreak. The potentially devastating effects if infection moves to a region where there are no restrictions means that the implementation of any such policy requires agreement from all stakeholders, in advance, of what level of risk is acceptable, weighed against the potential socio-economic benefits: it is therefore acknowledged that any epidemiological analysis is only one component of this decision process.

## Materials and Methods

The risk of escape from an RZ depends upon the number of IPs 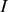, the expected transmission rate from an IP (as reflected by 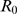, which encompasses factors such as local animal density and species) and, crucially, the distribution of distances at which such transmissions might occur (i.e. the transmission kernel).

### Transmission kernel

Spread of infection at various distances is likely to be by different routes, with direct contact or airborne transmission only possible over shorter distances and movement of fomites, persons or animals being primarily responsible for longer range transmissions. Often, it is not possible to differentiate the modes of transmission (even through post-epidemic tracing and analysis) and, furthermore, there will be a great deal of overlap between forms especially at the intermediate distances appropriate for RZs.

We therefore describe the relationship between distance and transmission using a probability-distribution function for the distance at which a transmission occurs without any assumptions about the mechanism of transmission (including whether restrictions are always adhered to or not): this does not describe a diffusive process but rather gives the probability of one or more infectious jumps occurring over a specific distance.

Records for the UK 2001 outbreak indicate that under movement controls most transmissions occur at a very local level (within a few km), with very few occurring beyond the range of any potential RZ [1]. Transmissions over much longer distances have been recorded, however, including airborne transmission over water beyond 200 km [16]. Consequently, we need to quantify the risk of long distance transmissions that are sufficiently rare for documented outbreaks to provide insufficient historical data. To allow for the inclusion of such low probability events we apply a fitted distribution, as opposed to applying historical data directly.

Here we use a simple two-parameter model function for the transmission kernel 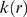 [17]. The probability distribution 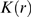 for the distance 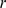 at which a given transmission occurs must be weighted by the potential number of farms at that distance, which we approximate as proportional to area: for a given kernel 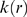 describing how the probability of transmission between individual farms scales with distance we thus set:
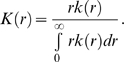



Parameters for the probability-distribution were derived by least-squares fitting to the recorded kernel data of [1]: this empirical kernel is the most appropriate since it separates transmission distance from farm type and size. Explicitly, the form of the kernel used is,
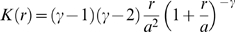
where 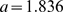 (95% confidence interval (CI): 1.624, 2.048) and 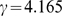 (95% CI: 3.932, 4.398); here 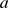 and 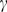 are the kernel off-set and power respectively and have been fitted to the data of [1]. The kernel is shown in Figure 3.

### Basic reproduction number

Estimates for 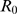 during the 2001 UK outbreak [18], [9], [19] are consistent with one another and indicate the values that can be expected: in the absence of movement restrictions (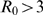); after imposition of a movement ban (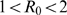); and following improved bio-security measures and possible “burn-out” [9] (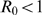). We would expect the second situation to apply initially in any future RZ. It is important to consider the virus strain in any estimate, although we note that the recent Surrey 2007 outbreak (with an expected airborne transmission potential greater than that of 2001) was contained with the mean 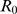 kept at similarly low values [4], [3]. This may well reflect changes in policy and procedure, as well as the scale of the outbreak, but indicates what might be reasonably expected in the future. Estimates of 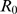 for a RZ - the mean reproduction ratio of all IPs in a RZ - are based on the number of infected holdings (rather than individual herd or flock sites), since this is used to define an IP.

### Model

Distance-kernels provide data on primary transmissions between two premises, but transmission over a given distance may occur in one or more stages. In particular, there may be undetected cases that may form intermediary steps between an IP and the boundary of an RZ. In cattle, the time between animals becoming infectious and showing clinical signs appears to be short [20], [21] so that good surveillance and efficient culling will make a sequence of multiple undetected IPs highly unlikely (in sheep signs are less obvious [22] but infectivity is also lower). Consequently, we consider only primary and secondary transmissions: that is, the risk of disease escaping the RZ directly from known IPs or via as yet undetected premises directly infected from a known IP. Under the imposition of movement restrictions the effects of secondary and subsequent transmissions on the overall likelihood of transmission over distances above a few kilometres are very small in practice, since the probability of one such transmission occurring, let alone multiple events, is already low.

For an IP with reproduction ratio R and transmission-distance probability-distribution 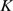 the expected transmission probability at 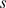 km is
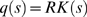
and the expected number of transmissions occurring above 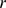 km is given by
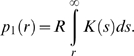



The probability of primary transmission from a circular RZ of radius 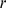 containing 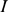 IPs at its centre is
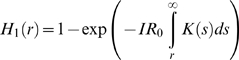
where 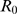 is the mean reproduction ratio for all IPs; it is assumed that all IPs have the same transmission distribution kernel. The expected number secondary transmissions outside the RZ from each IP is:
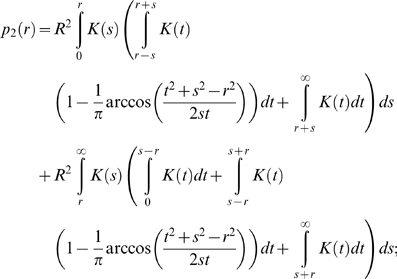
the first term refers to secondary transmissions outside the RZ from a primary transmission inside the RZ; the second to such transmissions occurring from a primary transmission which is already outside the RZ. The expression for 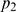 is derived by first considering a primary transmission occurring at point 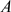 a distance 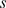 from the centre of the RZ, and then considering a secondary transmission occurring at a point 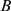 a distance 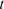 from 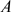. If 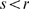 then 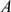 remains inside the RZ and we require 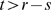 in order for 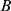 to be outside. By considering the possible location of 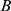 as a circle of radius 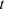 with centre 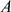, we note that 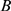 will always be outside the RZ if 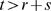. For 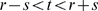 we need to calculate the probability that 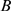 will land outside the RZ as opposed to inside, which, assuming all directions are equal, is equivalent to the proportion of the circle which is outside the original RZ disc with radius 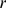. This fraction is given by 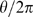 where 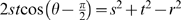. Finally we sum over all possible 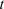, weighted by the transmission distance distribution 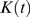, and over all possible 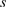, weighted by 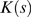. The calculation for secondary transmissions which occur from a point 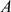 outside of the RZ (i.e. 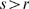) are similar, although now only if the 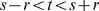 is there a possibility of 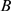 landing back inside the RZ: if 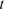 is smaller or larger the transmission can only under– or over-shoot the RZ respectively.

The hazard function for primary and secondary transmissions for an individual IP,
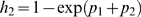
can then be extended to yield 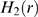 by considering 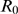 as above.

For primary transmission only the required RZ radius 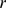 for a given hazard level 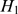 in terms of the number of IPs 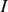 inside the zone and their mean reproduction ratio 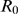 is the positive solution of:
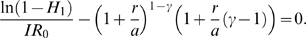



For secondary transmission this may be calculated numerically as a function of 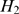.
